# Male-Biased Parasitism of Brandt’s Voles (*Lasiopodomys brandtii*) in Inner Mongolia, China

**DOI:** 10.3390/ani13081290

**Published:** 2023-04-09

**Authors:** Gaojian Li, Qinghe Wang, Min Zhang, Bin Hu, Shuyi Han, Chen Xiang, Guohui Yuan, Hongxuan He

**Affiliations:** 1 National Research Center for Wildlife-Borne Diseases, Institute of Zoology, Chinese Academy of Sciences, Beijing 100101, China; 2 University of Chinese Academy of Sciences, Beijing 100101, China; 3 Nanyang Wild Animals and Plants Protection Station, Nanyang 473000, China

**Keywords:** Brandt’s voles, sexual size dimorphism, intestinal parasites, male-biased parasitism, body size hypothesis

## Abstract

**Simple Summary:**

Previous studies had reported sex-biased parasitism (SBP) in small rodents. In this study, we investigate the prevalence of six intestinal parasites in Brandt’s voles (*Lasiopodomys brandtii*) that were captured in May, June, July, and August 2022 around the Xilingol Grassland in Inner Mongolia, China. *Syphacia obvelata*, *Aspiculuris tetraptera*, and the *Trichostrongylidae* family were the dominant intestinal parasites for the Brandt’s voles captured in the study areas. Season and human activities such as grazing had no significant effect on the infection rates of parasites, but the parasite reproduction level was higher when the ambient temperature was around 18 °C. We found that the sexual size dimorphism (SSD) was ubiquitous in Brandt’s voles, and males had bigger body sizes than females. Simple linear regression analysis showed a significant positive correlation between bodyweight and parasite infection rates, so the sex-biased parasitism in Brandt’s voles could be explained by the body size hypothesis, as a larger body could provide more ecological niches for parasitic infection.

**Abstract:**

The abundance and prevalence of parasitic infection often vary in different host sexes, and this phenomenon has been named sex-biased parasitism. Brandt’s voles are the dominant rodent species in typical steppe habitat and are widely distributed in Inner Mongolia, China, but the prevalence of parasites in Brandt’s voles are poorly reported. In this study, we investigated the prevalence of six intestinal parasites in Brandt’s voles in May, June, July, and August 2022 around the Xilingol Grassland in Inner Mongolia, China. The results showed that *Syphacia obvelata*, *Aspiculuris tetraptera*, and *Trichostrongylidae* family were the dominant intestinal parasites in Brandt’s voles that we captured in this study, and the infection rates of the three parasites were significantly higher in males than females, which showed obvious male-biased parasitism. Season and human activities such as grazing had no significant effect on the infection rates for different parasites, while the parasite reproduction level was higher when the ambient temperature was around 18 °C. Sexual size dimorphism was ubiquitous in Brandt’s voles, and it was mainly manifested by the differences in body weight and length between males and females. Simple linear regression analysis showed a significant positive correlation between bodyweight and parasite infection rates, so the sex-biased parasitism in Brandt’s voles could be explained by the body size hypothesis, as a larger body could provide more ecological niches for parasitic infection.

## 1. Introduction

Most host-parasite relationships are characterised by heterogeneity between the individuals in terms of the probability of contact, infection, and further transmission of the parasite or pathogen [1]. The individual heterogeneity results in a highly skewed distribution of parasite and host populations, and this phenomenon was named sex-biased parasitism [2]. Studies had reported that the prevalence and intensity of parasitism in vertebrates were often higher in males than females [3,4,5], and this had been named male-biased parasitism. On the other hand, female-biased parasitism [6] was also reported in the prevalence of parasites in invasive rodents from Chile [7] and wild bank voles (*Myodes glareolus*) [8]. Although sex-biased parasitism has different manifestations (female-biased or male-biased parasitism), the popular hypothesis is that the parasites will perform better within a male host [9]. 

The male reproductive success is strongly correlated with the competitive ability in mammals, which resulted in the evolution of large body size and weaponry [10]. As a consequence, polygynous mating systems in mammals are characterized by sexual size dimorphism, and males usually have a larger body size than females [11]. The divergent selections in morphology, physiology, life history, and behavior lead to sexual dimorphism in males and females; typically, most of the phenotypic variation between adults in a sexual population can be explained by sex differences [12]. As a result, the differences in parasite prevalence and clinical symptoms between males and females have been mainly attributed to the differentiated immune responses [13,14,15]. On the other hand, differences of morphology and hormone levels between males and females can impose selection on parasites, eventually leading to special parasitic adaptation to host sex, furthermore, the adaptation of parasites to the sex of their host can also shape the differential expression of parasite traits [16]. 

To explain the phenomenon of sex-biased parasitism, two hypotheses have been proposed, including the body size hypothesis and the sex-specific handicap hypothesis [2]. The body size hypothesis presumed that the host’s competitive ability was an important driving force for sex-biased parasitism and that the development of competitive ability could lead to bigger body size [11,17]. Thus, the larger hosts are expected to provide more ecological niches for parasitic infection. On the other hand, the males can trade off growth at the expense of immunity with limited energy, and low immunity can increase the risk of parasitic infection [18]. 

Sex-specific handicap hypothesis was proposed from the immunosuppressive effect caused by androgen production [19,20], and the testosterone could alter the allocation of limited resources between the development of ornamental traits and other tissues like the immune system [21,22]. It had been reported that the suppression of the immune system could enhance fertility [23]. In this case, males could increase mating rates by making themselves more attractive to females, and inhibit the immune responses for parasitic infection [19]. In particular, some studies on birds showed evidence for the immunosuppressive effect of testosterone [24,25,26]. Although there are hypotheses to explain sex-biased parasitism, the causes and consequences of sex-biased parasitism in the host-parasite research field remain a complex topic, and the mechanistic research of host-parasite interaction will benefit from more empirical studies across different parasitism systems [27]. 

Brandt’s voles (*Lasiopodomys brandtii*) are the dominant rodent species in the typical steppe habitat that extends from the central part of Inner Mongolia through the middle and east of Dornod Province, Republic of Mongolia, to the southern borders of Mongolia in Trans-Baikalia, Russia [28], and the distribution is discontinuous in Inner Mongolia, China [29,30]. In this study, we investigated the prevalence of six intestinal parasites (*Syphacia obvelata*, *Aspiculuris tetraptera*, *Trichostrongylidae* family, *Schizorchis ochotonae*, *Hymenolepis nana*, and *Echinostomatidae* family) in Brandt’s voles around Xilingol Grassland in Inner Mongolia, China. We found male-biased parasitism in the infection of *Syphacia obvelata*, *Aspiculuris tetraptera*, and the *Trichostrongylidae* family. The season and human activities such as grazing had no significant effect on the infection rates for different parasites. On the other hand, we also reported sexual sizes dimorphism in Brandt’s voles, and the males had bigger size (body weight and body length) than females. The simple linear regression analysis suggested a significant positive correlation between the host body weight and parasite infection rates, which indicated that the male-biased parasitism in Brandt’s voles can be explained by the body size hypothesis as a larger body could provide more ecological niches for parasitic infection. 

## 2. Materials and Methods

### 2.1. Study Area

**Figure 1:**
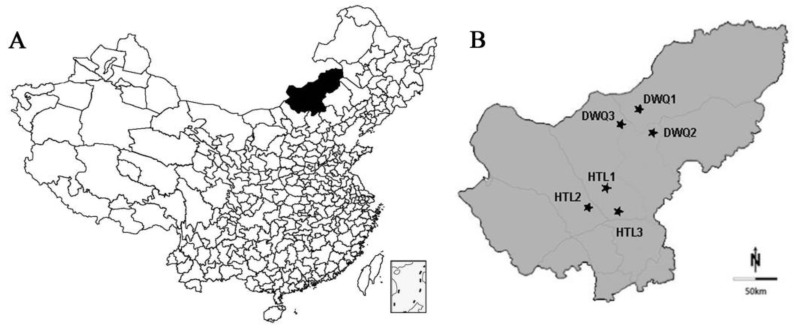
Study areas. (**A**) This study was carried out in Xilingol Grassland, Inner Mongolia, China (black background area). (**B**) The Brandt’s voles were captured from two discontinuous habitats, including Huitengliang (HTL, GPS reading 43°52′ N, 116°21′ E) and East Ujimqin (DWQ, GPS reading 46°16′ N, 117°83′ E) in Xilingol Grassland; each habitat included three trapping sites (ca. 20 km apart).

In this study, the Brandt’s voles were captured from two discontinuous habitats (Huitengliang, HTL, GPS reading 43°52′ N, 116°21′ E; and East Ujimqin, DWQ, GPS reading 46°16′ N, 117°83′ E) in Xilingol Grassland; each habitat included three sites for trapping (ca. 20 km apart). The areas for this study are presented in Figure 1. We captured Brandt’s voles in May, June, July, and August 2022. It should be noted that the trapping session coincided with the peak activity of Brandt’s voles [31] which could increase the success rate of capture. Peanuts were used as bait in trapping, and none of Brandt’s voles were hurt during the trapping process. Both the DWQ and HTL had a cold, semi-arid climate marked by long, cold, and very dry winters (from October to April next year). The annual precipitation is approximately 260 mm (10.2 in) in 2022, with more than half of it falling in July and August. The DWQ region had an open environment of rolling hills and exposed rocks, and we had been informed by the local populace that the grassland around here was forbidden for human grazing. While the HTL region had grazing activities for large livestock including cows, sheep and horses, which resulted in severe pasture degradation.

For the trapping of Brandt’s voles, the traps were set before 6 a.m. and collected after 8 a.m. in the morning. After trapping, the information of gender, body weight, length, and breeding status for each individual was recorded. The fecal samples were collected from the trap, and the fecal samples were discarded when there were two or more mice in the same trap. After trapping, the captured Brandt’s voles were transported to a lab for temporary feeding, and after the study, the mice were released in their habitats. The trap protocol followed the ASM guidelines [32] and the protocol for animal studies were approved by the Committee on the Ethics of Animal Experiments of the Institute of Zoology, Chinese Academy of Sciences (approval number: IOZ20220225-03). 

### 2.2. Parasite Detection

For parasite detection, the feces were collected from each mouse and stored at 4 °C for twelve hours on Petri dishes, and the feces were covered with damp blotting papers to standardize the humidity content. Parasite load was evaluated by the egg counting method with the fecal samples using a modified non-invasive McMaster’s method [33,34]. All samples were inspected under a microscope at a magnification of 100×. We used fecal egg counts (FEC, number of eggs per gram of feces) to reflect the overall intestinal parasite burden, and we calculated the infection rates to determine the prevalence for each parasite species. 

### 2.3. Statistical Analyses

We used the cubic spline (CS) method to explore the relationship between parasite burden and ambient temperature. The ambient temperature for different trapping sessions was retrieved from the website (https://lishi.tianqi.com/, accessed on 2 November 2022) and the average temperature for each day was recorded and used for statistical analysis. The cubic spline was performed in Python 3.7.9 with Scipy 1.7.3 package. The sample linear regression was used to explore the relationship between sexual size dimorphism and sex-biased parasitism or bodyweight and parasitic infection rate. Other statistical analysis methods are described in the figure legends. All calculations were performed with R 4.2.1 software [35] or GraphPad Prism 8 software (https://www.graphpad.com/, accessed on 2 March 2022). 

## 3. Results

### 3.1. Prevalence of the Six Intestinal Parasites in Brandt’s Voles

**Figure 2:**
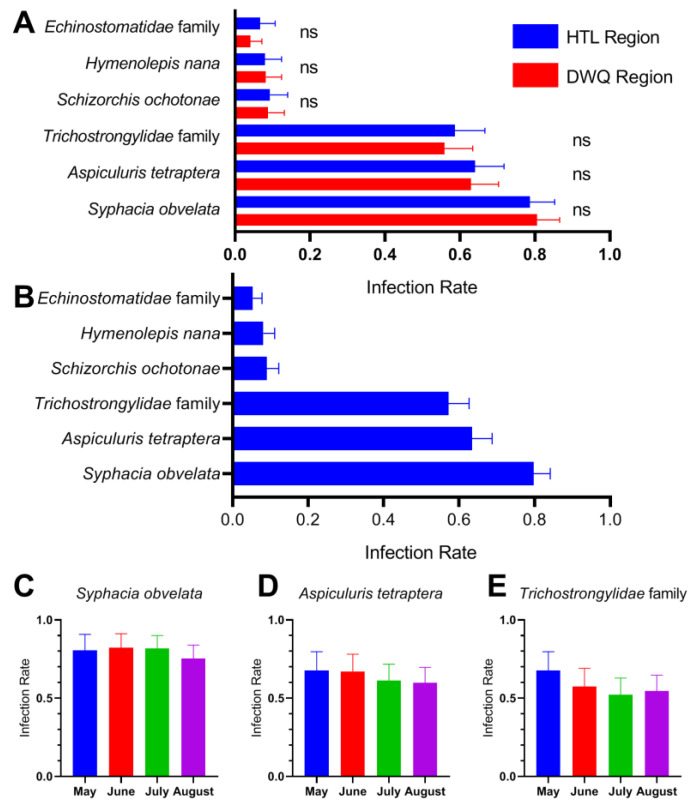
Season and human grazing had no significant effect on the infection rates for different intestinal parasites. (**A**) The infection rates of the six intestinal parasites in Brandt’s voles that were captured from DWQ and HTL. The grassland in DWQ was forbidden to human grazing, while the HTL region had the activities of large livestock and resulted in severe pasture degradation. (**B**) The overall infection rates of the six parasites in 320 Brandt’s voles. (**C**–**E**) The overall infection rates of the three parasites in May, June, July, and August 2022. The calculation of infection rates is presented as values ± 95% confidence intervals. An unpaired Student’s *t* test was used for statistical analysis, and a two-tailed *p* value was calculated. Statistical analysis was performed with GraphPad Prism 8 software; ns, non-significant.

In this study, we captured a total of 320 Brandt’s voles (sample size: DWQ1, *n* = 60; DWQ2, *n* = 62; DWQ3, *n* = 48; HTL1, *n* = 52; HTL2, *n* = 47; HTL3, *n* = 51) from two habitats (HTL and DWQ) in May, June, July, and August 2022. The infection rates of intestinal parasites, including *Syphacia obvelata*, *Aspiculuris tetraptera*, the *Trichostrongylidae* family, *Schizorchis ochotonae*, and *Echinostomatidae* family, were calculated, and the results were presented in Table 1. The infection rates for different parasites in DWQ and HTL were presented in Figure 2A and no significant differences were found between the two habitats. On the other hand, the overall infection rates of the parasites in 320 Brandt’s voles were presented in Figure 2B, which indicated that *Syphacia obvelata* (79.69%), *Aspiculuris tetraptera* (63.44%) and the *Trichostrongylidae* family (57.19%) were the dominant intestinal parasites. The infection rates of *Syphacia obvelata*, *Aspiculuris tetraptera*, and the *Trichostrongylidae* family in different seasons (May, June, July, and August) were shown in Figure 2C–E, respectively, and there was no statistical difference for the infection rates among the four months. These results indicated that the seasons and human grazing had no significant effect on the infection rates for these parasites. 

### 3.2. The Ambient Temperature Could Affect the Parasite Reproduction Level

**Figure 3:**
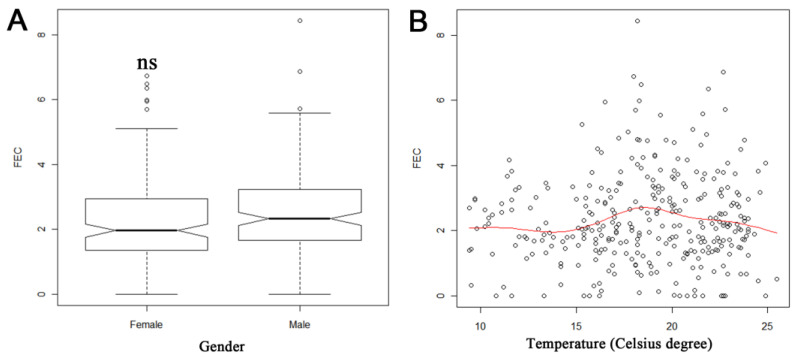
The parasite burden was related to ambient temperature. (**A**) The comparison of the overall FEC between males and females (An unpaired Student’s *t* test was used for significance analysis; ns, non-significant, *p* = 0.6565). All data are presented as means ± standard deviation, and the statistical analysis was performed with GraphPad Prism 8 software. (**B**) The cubic spline analysis method was used to explore the relationship between parasite burden and temperature the results indicated that when the ambient temperature was around 18 °C, the parasite would have a higher reproduction level.

We used the FEC (number of eggs per gram of feces) to reflect the parasite burden for each mouse. The FEC in different months (May, June, July, and August) for the two habitats (HTL and DWQ) were presented in Supplementary File S1 Figure S1 and no significant difference was found. Furthermore, we compared the overall FEC between males and females (Figure 3A) and also found no significant differences. On the other hand, we used the cubic spline method to explore the relationship between parasite burden and ambient temperature (Figure 3B), and the results indicated that when the ambient temperature was around 18 °C, the parasite would have a higher reproduction level. 

### 3.3. Sexual Size Dimorphism and Male-Biased Parasitism

**Figure 4:**
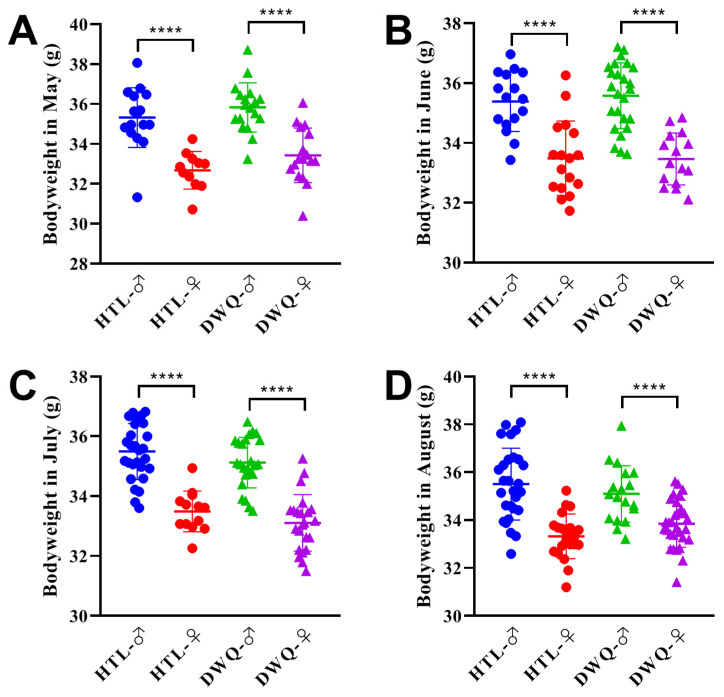
The comparison of bodyweight for male and female Brandt’s voles that captured in different months ((**A**), May; (**B**), June; (**C**), July; (**D**), August). The results indicated that the bodyweight of males was significantly higher than that of females. All data were presented as means ± standard deviation, and the data points were indicated. Unpaired Student’s *t* test was used for statistical analysis, and a two-tailed *p* value was calculated. The statistical analysis was performed with GraphPad Prism 8 software, **** *p*< 0.0001.

**Figure 5:**
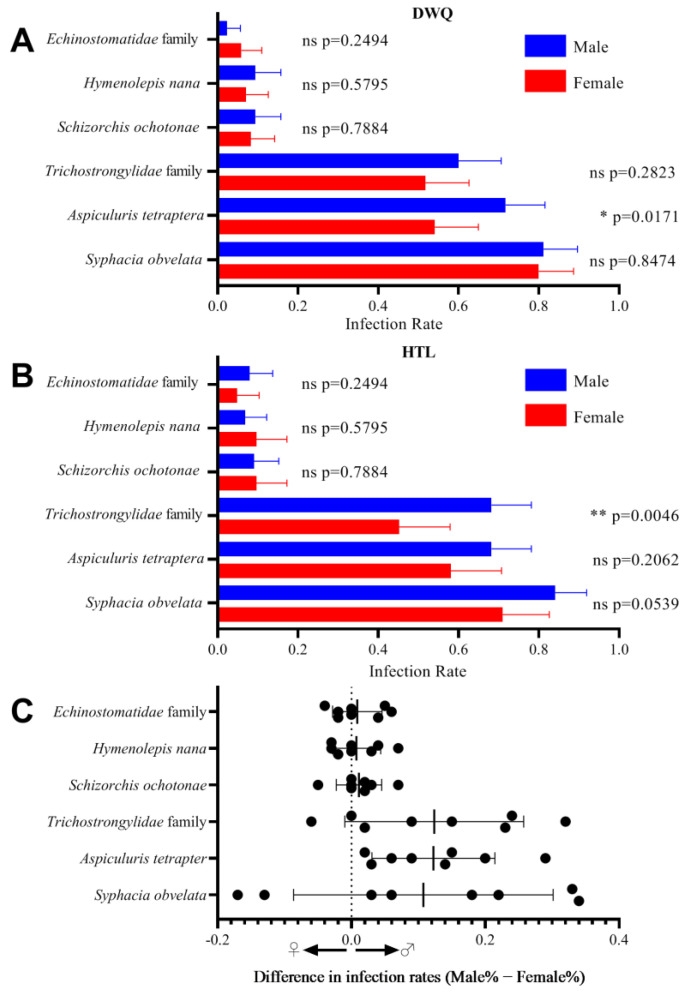
The comparison of parasitic infection rates between males and females for Brandt’s voles that were captured in the DWQ and HTL regions, showed obvious male-biased parasitism. (**A**) The infection rates for different parasite species in males and females that were captured in the DWQ region. (**B**) The infection rates for different parasite species in males and females that captured in the HTL region. (**C**) The differences in infection rates between males and females for the six parasite species. The calculation of infection rates is presented as values ± 95% confidence intervals. Unpaired Student’s *t* test was used to compare the differences in infectious status between males and females, two-tailed *p* value was calculated and presented; ns, non-significant differences, * *p* < 0.1, ** *p* < 0.01.

Body size is an important parameter to reflect the sexual size dimorphism. We compared the bodyweight and length between males and females, and the results are presented in Figure 4 and Supplementary File S1: Figure S2, respectively. The results indicated that Brandt’s voles showed obvious sexual size dimorphism, with males having a larger body size than females. The infection rates of different parasites in males and females were presented in Figure 5A,B. These results indicated that the infection rate of *Aspiculuris tetraptera* in males was significantly higher (*p* = 0.0171, *p* < 0.1) than that in females in the DWQ region, on the other hand, the infection rate of the *Trichostrongylidae* family in males was also significantly higher (*p* = 0.0046, *p* < 0.01) than that in females in the HTL region. Furthermore, the infection rates of *Syphacia obvelata*, *Aspiculuris tetraptera*, and the *Trichostrongylidae* family in males were higher than females. We calculated the differences in infection rates between males and females for the six parasites and presented them in Figure 5C, and the primary data was provided in Supplementary File S2. These results indicated obvious male-biased parasitism for the infection rates of *Syphacia obvelata*, *Aspiculuris tetraptera*, and the *Trichostrongylidae* family. 

### 3.4. Simple Linear Regression Analysis

**Figure 6:**
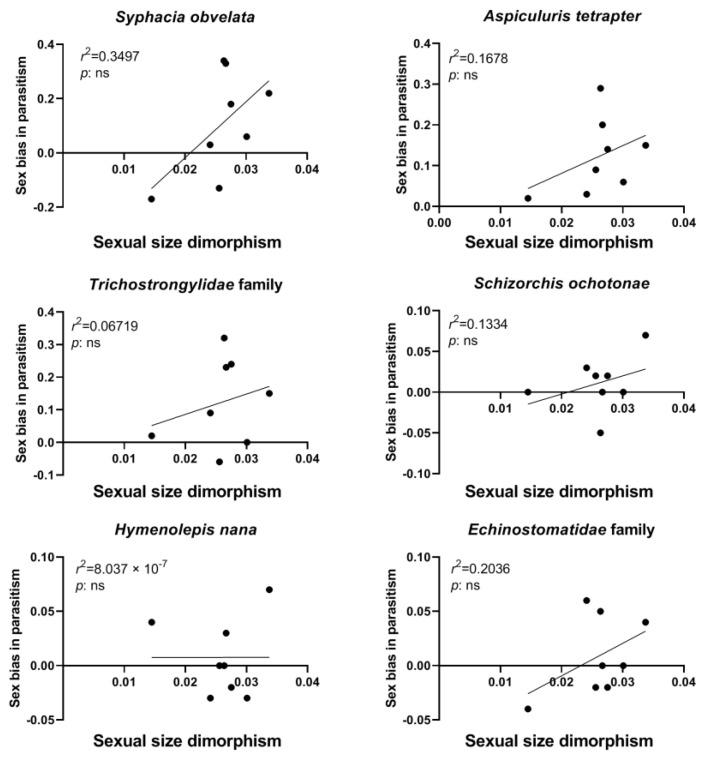
The simple linear regression analysis for sex-biased parasitism. The raw data for statistical analysis was presented in Supplementary File S2, the total data was grouped by region (DWQ and HTL) and month (May, June, July and August), the sexual size dimorphism and sex-biased parasitism for each group were calculated and presented. The simple linear regression analysis was performed with GraphPad Prism 8 software, and the R squared and *p* value were presented.

We used the following formula to represent the sexual size dimorphism (SSD): SSD = log_10_(mean male body weight/mean female body weight) [2], on the other hand, the sex-biased parasitism (SBP) was represented as SBP = male infection rate − female infection rate. Simple linear regression analysis was used to explore the relationship between sexual size dimorphism and sex-biased parasitism for the six intestinal parasites the results were presented in Figure 6, and the primary data is provided in Supplementary File S2. The results indicated that all six parasite species except *Hymenolepis nana* showed male-biased parasitism. On the other hand, the *p* values of the simple linear regression analysis in these parasites were all non-significant, and this could be due to the insufficient data volume. 

### 3.5. The Parasite Infection Rate Is Positively Correlated with Host Bodyweight

**Figure 7:**
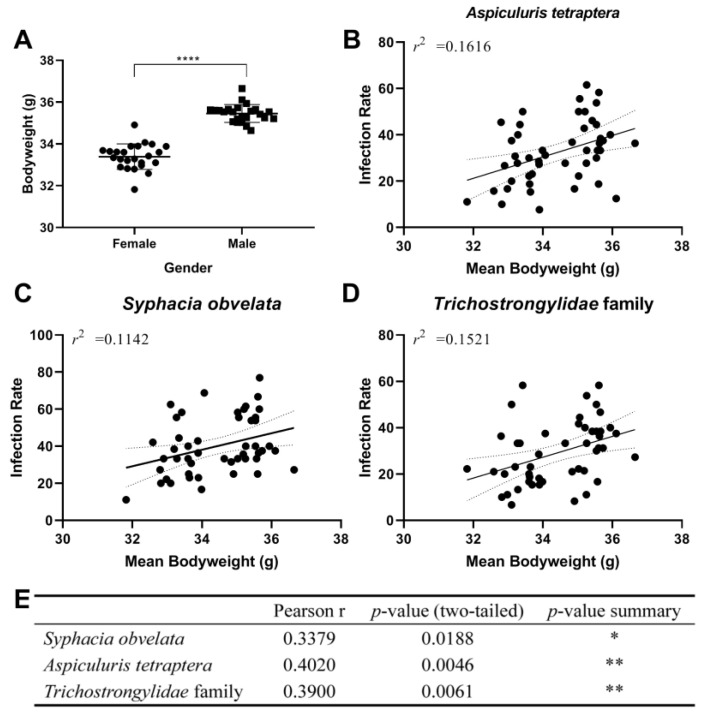
The simple linear regression analysis for the evaluation of the correlation between bodyweight and parasitic infection rate. (**A**) The comparison of bodyweight between males and females for Brandt’s voles captured in this study. Unpaired Student’s *t* test was used for statistical analysis, and the two-tailed *p* value was calculated, ****, *p* < 0.0001. (**B**–**D**) The simple linear regression analysis for the correlation between bodyweight and *Syphacia obvelata*, *Aspiculuris tetraptera*, and the *Trichostrongylidae* family infection rates. The raw data for statistical analysis was presented in Supplementary File S3, the data was grouped by month and location. In each group, the infection rates for different parasites and mean body weight were calculated and presented. The R square for each regression was presented, and the 95% confidence interval for each regression curve was indicated. (**E**) The Pearson correlation coefficient analysis results for the correlation between bodyweight and parasitic infection rates. The simple linear regression analysis and Pearson correlation coefficient were performed with GraphPad Prism 8 software; * *p* < 0.1, ** *p* < 0.01.

We compared the differences in body weight between males and females in the 320 Brandt’s voles (Figure 7A), and the result indicated that the body weight of males was significantly higher than that of females (*p* < 0.0001). We used the prevalence of *Syphacia obvelata*, *Aspiculuris tetraptera*, and the *Trichostrongylidae* family for statistical analysis as these parasites had higher infection rates. A simple linear regression analysis was used to explore the relationship between bodyweight (mean body weight of Brandt’s voles in different sites and months) and parasitic infection rates of *Syphacia obvelata*, *Aspiculuris tetraptera*, and the *Trichostrongylidae* family. The raw data for simple linear regression analysis was provided in Supplementary File S3, and the results are presented in Figure 7B–D, respectively. We also used the Pearson correlation coefficient to test the correlation between body weight and parasitic infection rate, and the results are presented in Figure 7E. These results showed a significant positive correlation between body weight and parasite infection rates, so the male-biased parasitism could be explained by the body size hypothesis, as a larger body could provide more ecological niches for parasitic infection. 

## 4. Discussion

Although Brandt’s voles have a wide distribution, the prevalence of their parasites are poorly studied [36,37]. This study focused on the prevalence of intestinal parasites, because their infections were very common in small rodents, which made them very suitable for long-term follow-up investigation. Intestinal nematode infection hads been reported to affect host fitness and mortality in wild animal populations [38,39]. For example, the infection of *Heligmosomoides polygyrus* has been found to regulate population size in the laboratory [40]. The calculation of infection rates indicated that the *Syphacia obvelata*, *Aspiculuris tetraptera*, and the *Trichostrongylidae* family are the dominant intestinal parasites in Brandt’s voles with high infection rates (Figure 2). This might be because these parasites had a simple life cycle and a short incubation period; furthermore, Brandt’s vole are social animals, which can increase the transmission of these parasites [41]. The infection rates of other parasites, including *Schizorchis ochotonae*, *Hymenolepis nana*, and the *Echinostomatidae* family, were very low; this might be because the grassland environment lacked the corresponding intermediate hosts. 

The trapping sessions in this study were consistent with the period of reproductive peak of Brandt’s voles. During the breeding period, males can produce high levels of androgen, which can lead to immunosuppression and increase the risk of parasitic infection [19]. Studies have reported that there are sex differences in immunity and susceptibility to parasites in a variety of mammals, with males generally having a lower immunity and being more sensitive to parasites [42]. While in this study, the level of androgen was not detected, and this could be our future work. 

Although there are differences in immune responses to parasitic infection between males and females due to gender and hormonal effects on the immune system, different behaviors, home ranges, and/or diets can also lead to differences in exposure to parasites. Based on this, the animal behavior factor can also be responsible for sex-biased parasitism, and the mechanisms of the encounter filter have been reported [43]. In promiscuous or polygynous mammals, males are more mobile than females, and different males have a larger, wider overlap range [44]. Higher mobility and greater home ranges allow males to increase mating opportunities. However, this also increases their risk of parasitism. In addition, the higher overlap between males’ home ranges also increases their chances of parasite exchange, thus increasing the average species richness of parasite combinations [13]. 

Obvious sexual size dimorphism in Brandt’s voles was found and males had bigger body sizes (body weight and length) than females. The results of simple linear regression analysis suggested a significant correlation between bodyweight and parasite infection rates (Figure 6 and Figure 7). Based on the results of simple linear regression analysis, the male-biased parasitism in Brandt’s voles could be explained by the body size hypothesis, as a larger body could provide more ecological niches for parasitic infection. Throughout, we are focusing on the role of body mass in researching the driving force behind sex-biased parasitism, but the mechanism by which body mass controls sex-biased parasitism is unclear. One possibility is that size-dependent resource requirements occur. For example, male wood mice have larger home range sizes and greater mobility than females [45], so they have an increased probability of encountering questing ticks. Another possibility is that size dependent behavior occurs. Males may spend less time indulging in self-maintenance, such as grooming in favor of searching for and competing for females. Future work should combine these factors with models in order to gain a full understanding of the mechanisms that underpin male-biased parasitism. 

## 5. Conclusions

In this study, we investigated the prevalence of six intestinal parasites in Brandt’s voles around the Xilingol Grassland in Inner Mongolia, China, in May, June, July, and August 2022. *Syphacia obvelata*, *Aspiculuris tetraptera*, and the *Trichostrongylidae* family were the dominant intestinal parasites in Brandt’s voles with high infection rates. Season and human activities such as grazing had no significant effect on the infection rates for different parasites, but the parasite would have a higher reproduction level when the ambient temperature was around 18 °C. The differences in infection rates of *Aspiculuris tetraptera* and the *Trichostrongylidae* family between males and females were statistically significant. The sexual size dimorphism was ubiquitous in Brandt’s voles, and the parasite infection rate had a significant correlation with bodyweight, so the male-biased parasitism could be explained by the body size hypothesis, as a larger body could provide more ecological niches for parasitic infection. 

## Figures and Tables

**Table 1:** The infection rates of different parasites in male and female Brandt’s voles captured from DWQ and HTL regions in May, June, July and August, 2022.

Area	Time	Number	Gender	Number	Infection Rate					
					*Syphacia obvelata*	*Aspiculuris tetraptera*	*Trichostrongylidae* Family	*Schizorchis ochotonae*	*Hymenolepis nana*	*Echinostomatidae* Family
DWQ	May	35	♀	17	13 (76.47%)	13 (76.47%)	12 (70.59%)	2 (11.76%)	2 (11.76%)	1 (5.88%)
			♂	18	15 (83.33%)	15 (83.33%)	12 (66.67%)	2 (11.11%)	1 (5.56%)	1 (5.56%)
	June	40	♀	15	10 (66.67%)	9 (60%)	7 (46.67%)	4 (26.67%)	0 (0%)	1 (6.67%)
			♂	25	23 (92%)	17 (68%)	16 (64%)	4 (16%)	1 (4%)	1 (4%)
	July	47	♀	23	22 (95.65%)	11 (47.83%)	12 (52.17%)	0 (0%)	3 (13.04%)	1 (4.35%)
			♂	24	16 (66.67%)	15 (62.5%)	9 (37.5%)	1 (4.17%)	3 (12.5%)	0 (0%)
	August	48	♀	30	23 (76.67%)	13 (43.33%)	13 (43.33%)	1 (3.33%)	1 (3.33%)	2 (6.67%)
			♂	18	15 (83.33%)	14 (77.78%)	14 (77.78%)	1 (5.56%)	3 (16.67%)	0 (0%)
HTL	May	27	♀	11	8 (72.73%)	5 (45.45%)	7 (63.64%)	0 (0%)	0 (0%)	1 (9.09%)
			♂	16	14 (87.5%)	9 (56.25%)	11 (68.75%)	2 (12.5%)	2 (12.5%)	2 (12.5%)
	June	33	♀	17	13 (76.47%)	11 (64.71%)	8 (47.06%)	2 (11.76%)	2 (11.76%)	0 (0%)
			♂	16	14 (87.5%)	12 (75%)	11 (68.75%)	3 (18.75%)	1 (6.25%)	2 (12.5%)
	July	41	♀	13	10 (76.92%)	8 (61.54%)	6 (46.15%)	2 (15.38%)	1 (7.69%)	0 (0%)
			♂	28	24 (85.71%)	20 (71.43%)	19 (67.86%)	0 (0%)	1 (3.57%)	2 (7.14%)
	August	49	♀	21	13 (61.9%)	12 (57.14%)	7 (33.33%)	2 (9.52%)	3 (14.29%)	2 (9.52%)
			♂	28	22 (78.57%)	19 (67.86%)	19 (67.86%)	3 (10.71%)	2 (7.14%)	1 (3.57%)

## Data Availability

The original data presented in the study are included in the article; further inquiries can be directed to the corresponding author.

## Supplementary Materials

The following supporting information can be downloaded at: https://www.mdpi.com/article/10.3390/ani13081290/s1, Supplementary File S1, the FEC results in different months for the two habitats (DWQ and HTL) were presented in Figure S1; the comparison of body height for male and female Brandt’s voles captured in different months were presented in Figure S2. Supplementary File S2, the raw data used in simple linear regression analysis for sex-biased parasitism. Supplementary File S3, the raw data for simple linear regression analysis for the correlation between bodyweight and the infection rates of *Syphacia obvelata*, *Aspiculuris tetraptera*, and the *Trichostrongylidae* family.
