# Supplementary material for: Male-Biased Parasitism of Brandt’s Voles (Lasiopodomys brandtii) in Inner Mongolia, China
Source: Animals (Basel). 2023 Apr 9;13(8):1290. doi: 10.3390/ani13081290 (PMC10135223; doi:10.3390/ani13081290)
Supplement: Supplementary file 1 [file animals-13-01290-s001.zip › Supplementary File S1.pdf]

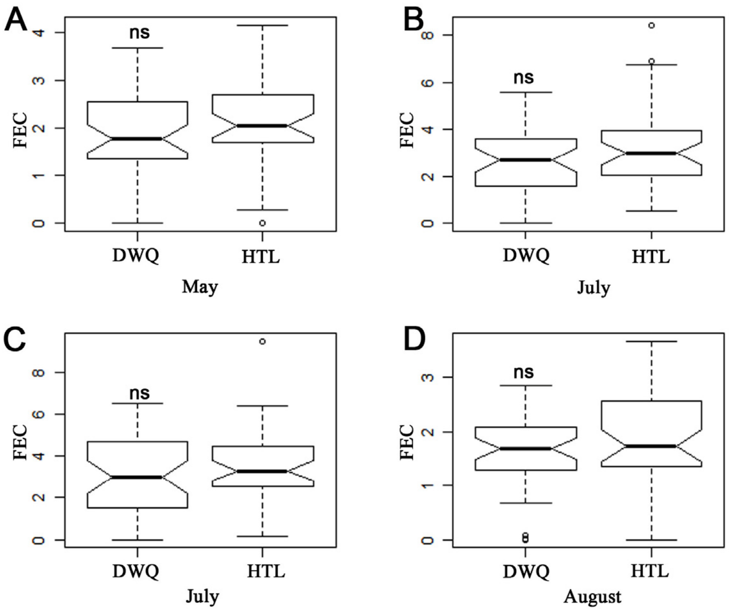

Figure S1. The FEC (Fecal egg counts) results in different months (May, June, July and August) for the two habitats (HTL and DWQ). The FEC results in different months showed non significant differences in DWQ and HTL regions. Unpaired Student *t* test was used for significance analysis; ns, non significant differences. ns, non-significant.

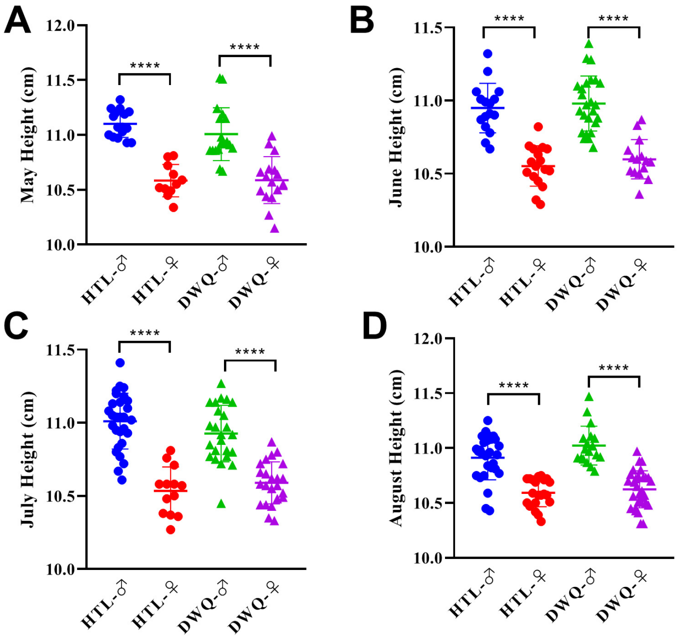

Figure S2. The comparison of body height for male and female Brandt's voles captured in different months. The results indicated that the body height of males were significantly higher than that in females. The data were presented as means ± standard deviation and every data point was labeled. Unpaired Student *t* test was used for significance analysis, **** $p < 0.0001$.
